# Abdominal adiposity is the main determinant of the C-reactive response to injury in subjects undergoing inguinal hernia repair

**DOI:** 10.1186/1476-9255-10-5

**Published:** 2013-02-07

**Authors:** Sashidhar Irkulla, Bedri Ujam, David Gaze, Devinder Kumar, Michael A Mendall

**Affiliations:** 1 Croydon University Hospital, Mayday Rd, Thornton Heath, Surrey, CR7 7YE, UK; 2 St George’s Medical School, London, UK

**Keywords:** Inflammation, C-reactive protein, Waist: hip ratio, Injury

## Abstract

**Background:**

Obesity and serum C-reactive protein (CRP) (a sensitive marker of inflammatory activity) are associated with most chronic diseases. Abdominal adiposity along with age is the strongest determinant of baseline CRP levels in healthy subjects. The mechanism of the association of serum CRP with disease is uncertain. We hypothesized that baseline serum CRP is a marker of inflammatory responsiveness to injury and that abdominal adiposity is the main determinant of this responsiveness. We studied the effect of abdominal adiposity, age and other environmental risk factors for chronic disease on the CRP response to a standardised surgical insult, unilateral hernia repair to not only test this hypothesis but to inform the factors which must be taken into account when assessing systemic inflammatory responses to surgery.

**Methods:**

102 male subjects aged 24-94 underwent unilateral hernia repair by a single operator. CRP was measured at 0, 6, 24 and 48 hrs. Response was defined as the peak CRP adjusted for baseline CRP.

**Results:**

Age and waist:hip ratio (WHR) were associated both with basal CRP and CRP response with similar effect sizes after adjustment for a wide-range of covariates. The adjusted proportional difference in CRP response per 10% increase in WHR was 1.50 (1.17-1.91) p = 0.0014 and 1.15(1.00-1.31) p = 0.05 per decade increase in age. There was no evidence of important effects of other environmental cardiovascular risk factors on CRP response.

**Conclusion:**

Waist:hip ratio and age need to be considered when studying the inflammatory response to surgery. The finding that age and waist:hip ratio influence baseline and post-operative CRP levels to a similar extent suggests that baseline CRP is a measure of inflammatory responsiveness to casual stimuli and that higher age and obesity modulate the generic excitability of the inflammatory system leading to both higher baseline CRP and higher CRP response to surgery. The mechanism for the association of baseline CRP and waist:hip ratio to chronic disease outcomes could be through this increase in inflammatory system excitability.

## Introduction

Obesity is associated with an increased risk of developing and mortality from many chronic diseases including atherosclerosis, most forms of epithelial cancer and other non-vascular diseases [1-3]. Likewise serum levels of the marker of acute inflammation C-reactive protein (CRP) in otherwise healthy subjects are associated with a similar range of diseases and outcomes but also include smoking related respiratory disease [4]. Obesity and in particular abdominal adiposity along with age are the strongest determinants of baseline CRP levels in healthy subjects with other environmental and behavioural risk factors for disease having a weaker but measureable effect on levels [5-8].

As yet the mechanism linking circulating levels of CRP within the reference range to the pathogenesis of atherosclerosis and other chronic disease is uncertain. CRP itself is unlikely to be of pathogenic significance [9,10]. It has been proposed variously that levels reflect increases in inflammation which change cardiovascular risk factors or that inflammatory mediators directly promote disease or that CRP levels are an epiphenomenon of tissue stress due to the injury which mediates disease [7,11,12].

We postulated that basal CRP levels are in fact a measure of the individual’s inflammatory responsiveness to the environment. Hence the explanation for the association between both obesity and CRP with a range of chronic disease outcomes could be because of the effect of obesity on an individual’s inflammatory responsiveness. These inflammatory mechanisms mediate the effect of the environment on chronic disease a various disease locations eg the blood vessel wall in atherosclerosis secondary to the effects of hypertension and various organ locations in cancer.

Host characteristics which affect the inflammatory response to injury have been little studied, although in a small study the inflammatory response was reported to be higher in obese subjects undergoing laparoscopic cholecystectomy [13]. One way to do this would be study the response to an easily standardisable insult administered to otherwise healthy subjects. We postulated that abdominal adiposity, the major determinant along with age of basal CRP levels would be associated with increased response.

Our primary aim was to determine whether abdominal adiposity was related to post insult CRP levels following a standardised surgical insult. The secondary aim was to determine what effect other environmental risk factors for chronic disease had on the CRP response. The standardised insult we chose was unilateral hernia repair.

## Methods

Following approval by the local research and ethics committee, and subject to informed consent, patients were recruited consequently. All male patients aged greater than 16 undergoing unilateral primary open inguinal hernia repair by a single operator between December 2007 and March 2009 were included. All subjects underwent a general anaesthetic. Subjects were excluded if they had diabetes, chronic renal or cardiac failure, on long term steroid or NSAID therapy (apart from cardioprotective aspirin), if they were otherwise unfit for surgery or if they were unable to give informed consent.

Base line measurements including heart rate, blood pressure, height, weight, waist and hip circumferences were recorded on the day of surgery. Waist measurements were taken at the midpoint between the lower ribs and iliac crest and hip measurements as the maximum circumference between the waist and upper thigh. Patient information including age, race, past medical history, drug history, smoking habits, physical activity and occupational history was collected at the time of recruitment. Drug history was taken for any medication consumed in the previous week (NSAIDs and anti-hypertensive medication). Subjects were said to be hypertensive if currently taking anti-hypertensive medication. Post operative data were also collected regarding wound infection and the presence of systemic inflammatory response syndrome but no cases of either were observed.

All were operated on by a single senior surgeon using Lichtenstein mesh repair with proline. A standardized general anaesthetic technique was used. All patients received 20 ml of 0.25% chirocaine wound infiltration; a standardized oral analgesic (Co-codamol) was given to patients on discharge. All operations were performed in the morning and patients were discharged at 6 hours post surgery. The baseline blood test was collected on arrival for the procedure and the 6 hour specimen soon before discharge home. The 24 and 48 hr specimens were obtained by visiting the patients in their home the morning of the following 2 days to within +/- 1.5 hour of the time of their operation with no requirement for fasting.

Serum C-reactive protein was determined using a high sensitivity near infrared particle immunoassay rate method on a LX-20pro (Beckman Coulter Inc, High Wycombe, UK) according to the manufacturer’s instructions. The sample is incubated with an anti-CRP antibody coated to a micro particle to form a [CRP (sample)-antibody complex] causing turbidity in the reaction. The change in absorbance is measured specrophotometrically at 940 nm and is directly proportional to the concentration of CRP. The assay has a measuring range of 0.2 – 80 mg/L. The functional sensitivity (20% coefficient of variation) was <0.18 mg/L. The total assay precision as reported by the manufacture was 3.4-5.1% at 0.7 – 77.5 mg/L. The reported 95th centile reference interval based on 551 healthy non smoking adults provided by the manufacturer (>18 years of age) was 0 to 7.48 mg/L.

### Statistical methods

CRP response to surgery was defined as the postoperative CRP value adjusted for the baseline CRP in ANOVA models or regression models when only continuous or 2 level explanatory variables were included. Thus individual CRP responses correspond to the model adjusted postoperative CRP values as if each patient had the same mean preoperative CRP value. As the postoperative CRP values did not always peak at 48 hours, sometimes earlier (especially in younger patients) the highest of the 6, 24 and 48 hour values, the peak values were considered as the primary outcome in statistical modelling. If any post-operative values were missing, the highest of the timepoints for which there was data was taken. A secondary measure of response was also included consisting of the difference between peak and baseline CRP log transformed.

The following variables were included in multivariate ANOVA and linear regression models: age at surgery, CRP before surgery, peak CRP after surgery, ethnic group (Caucasian, Afro-carribean, Asian or other), exercise (binary, none or exercise less than twice a week versus any exercise twice a week or more), smoking (current, ex and never), waist:hip ratio, occupation (manual, non-manual, retired), duration of surgery (minutes), wound length (cm). Each interval-scale variable was considered either on its original scale (age, wound length, duration of surgery) or after logarithmatic transformation (waist:hip. C-reactive protein at each time point ) to normalise the distribution. Not more than one of the obesity markers BMI, or waist:hip ratio were included in the models. Interactions were not considered.

Two multivariate models are presented: firstly a model including all explanatory variables and secondly a model including only waist:hip, age and duration of surgery. Finally, to allay any fears over confounding, the relationship of waist:hip ratio to the other explanatory variables is presented.

Statvew SE was used for the statistical analysis.

### Sample size calculations

Based on initial pilot study in 40 subjects, the correlation coefficient for waist hip with log10 of the mean CRP at 24 hours was 0.375. The standard deviation of waist hip ratio was estimated to be 0.08 and the standard deviation of the log of the mean CRP at 24 and 48 hrs would be 1.13 as would the standard deviation of the maximum CRP at either 24 or 48 hrs. A sample size of 90 was calculated as giving a power of 80% to detect a 1.8 fold ratio of CRP across the interquartile range ie the CRP at the 75th quartile would be 1.8 times that of the 25th centile.

## Results

**Figure 1:**
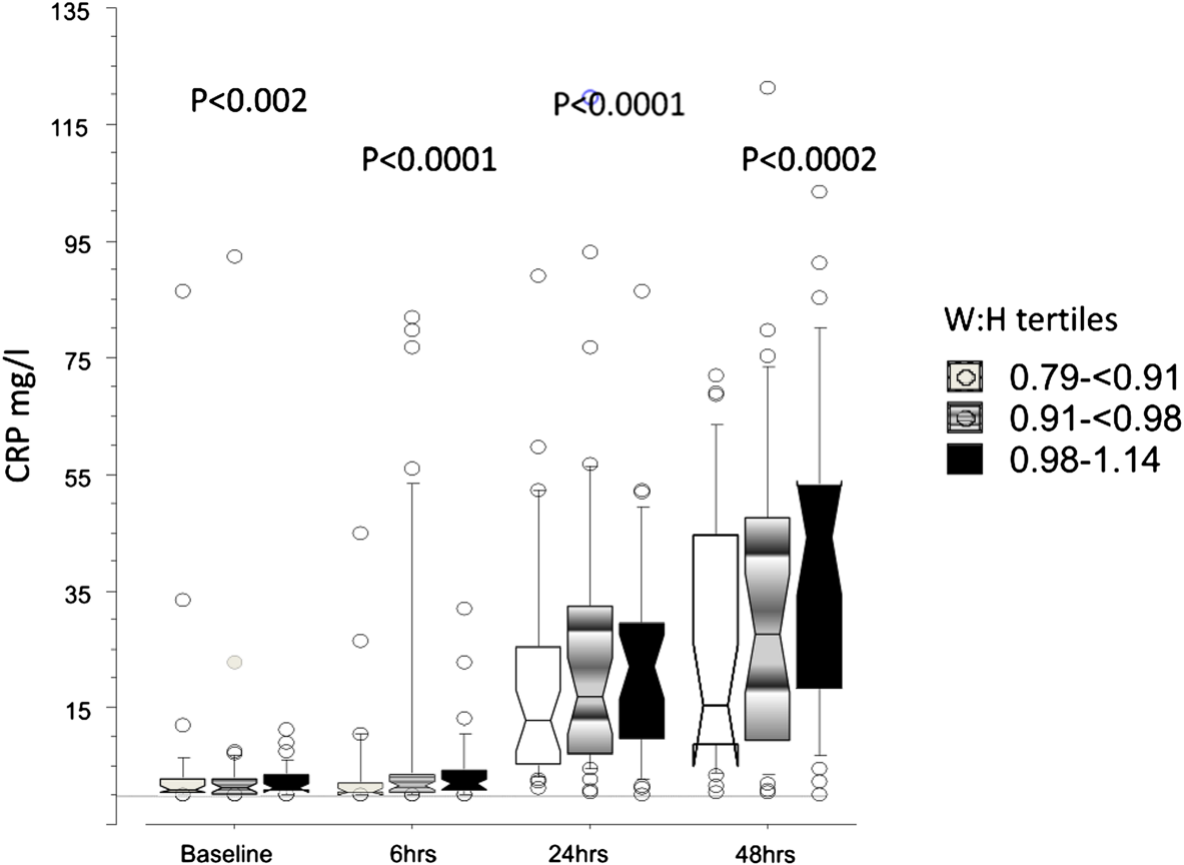
A box and whisker plot of the association of waist:hip ratio by tertile and CRP at different time points.

Table 1 shows the baseline characteristics of the 102 subjects, CRP levels at different time points together with operative details. There were no cases of post-operative wound infection. Table 2 displays the univariate analysis of the association of baseline characteristics with baseline CRP, peak CRP post surgery and peak CRP-baseline CRP. Figure 1 shows the association of waist:hip ratio with post operative CRP by wasit:hip ratio tertile at different time points. Significant associations existed between age, waist circumference and waist:hip ratio with baseline, post operative CRP and change in CRP. BMI demonstrated associations in the expected directions but these were not significant. Duration of surgery was associated with all three CRP measures, although the relationship with baseline CRP was of borderline statistical significance. Wound length likewise was associated with baseline and peak CRP but not additive change. Smoking status and social class were associated with baseline CRP but not post-operative CRP. However social class was associated with difference in CRP. Exercise history demonstrated associations in the expected direction for baseline CRP but this was not significant. Anti-hypertensive treatment was associated with post operative CRP and change in CRP

**Table 1:** Baseline characteristics and c-reactive protein at different time points of study subjects

	**Characteristics**
Age (mean, sd, range)	60.6 (14.4, 24-90)
Ethnic Group: n, %	
White Caucasian	77 (76%)
Afro Carib	14 (14%)
Asian	6 (5%)
Other	5 (5%)
Smoking status (%)	
Current	21 (21%
Ex	31 (30%)
Never	50 (49%)
Anti-hypertensive medication	21 (20%)
Waist:hip ratio (mean, sd,range)	0.945 (0.071, 0.79-1.14)
Waist circumference (cm) (mena, sd, range)	94.9 (9.54, 74-127)
BMI (mean, sd, range)	25.7 (3.09, 17.9-37)
Exercise	
<2 x /week	68 (67%)
2 + x/week	34 (33%)
Occupation:	
Manual	19 (19%)
Non-manual	34 (33%)
retired	49 (48%)
Wound length cm mean, sem, range	8.43 (0.84, 7-10)
Duration of surgery mins	18.2 (5.54, 8-40)
Mean, sd, range	
Serum C-reactive protein, geom. Mean, median, range) at baseline n = 102	1.28 (1.34, 0.1-92.4)
Serum C-reactive protein, geom. Mean, median, range) at 6 hrs n = 95	1.22 (1.30, 0.1-79.8)
Serum C-reactive protein, geom. Mean, median, range) at 24 hrs n = 98	13.7 (17.6, 0.8-120)
Serum C-reactive protein, geom. Mean, median, range) at 48 hrs n = 97	20.0 (27.4, 0.1-121.4)
Serum C-reactive protein, geom. Mean, median, range) peak n = 102	24.0 (28.4, 0.1-121.4)

**Table 2:** Relation of baseline risk factors to baseline CRP, peak post-operative CRP and absolute change in CRP following surgery

	**Baseline CRP (proportional change)**	**Peak CRP (proportional change)**	**Difference in peak CRP peak and baseline(proportional change)**
Baseline CRP (per 10 fold increase)		2.44 (1.76-3.38) p < 0.0001	1.23 (0.86-1.76) ns
Waist:hip ratio (per 10% increase)	1.70 (1.22-2.37) p = 0.0022	1.86 (1.44-2.40) p < 0.0001	1.52 (1.17-1.98) p = 0.0019
BMI(per unit change)	1.06 (0.97-1.16) p = 0.16	1.05 (0.97-1.12) p = 0.24	1.06 (0.99-1.14) p = 0.1
Waist circumference (per 10 cm increase)	1.68 (1.28- 2.20) p = 0.0002	1.60 (1.29- 1.98)	1.43 (1.15- 1.77) p = 0.0015
		P < 0.0001	
Weight (per 10 kg increase)	1.21 (0.95-1.52)	1.14 (0.95-1.39)	1.16 (0.97-1.39) p = 0.09
Age (per decade)	1.38 (1.15-1.66) p = 0.0006	1.32 (1.14-1.52) p = 0.0003	1.18 (1.02-1.37) p = 0.02
Duration of surgery (per minute)	1.05 (1.01-1.11) p = 0.04	1.06 (1.02-1.10) p = 0.003	1.05 (1.02-1.09) p = 0.007
Wound length (per cm)	1.46 (1.06-2.00) p = 0.02	1.33 (1.03-1.73) p = 0.03	1.28 (0.99-1.65) p = 0.06
*Ethnic Group: n, %			
White Caucasian	1.46 (1.00-2.63)	1.38 (0.91- 1.51)	1.17 (0.78- 1.74)
Afro Carib	1.27 (0.66- 2.43)	0.83 (0.55- 1.50)	0.87 (0.50- 1.73)
Asian	1.67 (0.73- 3.84)	1.15 (0.55- 2.40)	1.12 (0.54- 2.30)
Other (ref)	0.29 P = 0.06	0.75	0.88
*Smoking status (%)			
Current (reference)	0.80	0.87	0.93 (0.70-1.24)
Ex	1.66 (1,12- 2.45)	1.30 (0.95- 1,78)	1.13 (0.82- 1.56)
Never	0.76 (0.53- 1.08) P = 0.04	0.88 (0.66- 1.17)	0.95
Exercise			
<2 × /week (reference)			
2 + ×/week	0.64 (0.36-1.13) p = 0.12	0.95 (0.59-1.52)	1.14 (0.72-1.79)
*Occupation			
Manual (reference)	0.73 (0.47- 1.14)	0.68 (0.48- 0.96)	0.69 (0.48- 0.99)
Non-manual	0.80 (0.54- 1.17)	1.06 (0.78- 1.43)	1.18 (0.87- 1.60)
retired	1.73 p = 0.007	1.39 P = 0.1	1.23 P = 0.002
Anti-hypertensive medication	1.63 (0.86-3.10)	1.91 (1.13-2.61) p = 0.02	1.82 (1,10-2.61) P = 0.02

CRP was log transformed for the purposes of analysis of baseline CRP and post-operative CRP and the absolute difference in pre-op and post-operative CRP was log transformed after adding 1 to the difference.

*ANOVA coefficients, statistical significance determined by ANOVA.

Table 3 displays the multivariate analysis of the associations of baseline and peak CRP and additive change in CRP including all the baseline predictor variables with the exception of BMI, and waist circumference. Strikingly, waist:hip ratio was related to all CRP measures. Baseline CRP was related to peak CRP but not the additive change. The magnitude of the effect was similar for baseline, peak and additive change in CRP. Although age demonstrated a similar magnitue of effect for the three CRP measures, the effect was only significant for peak CRP. Duration of surgery was associated strongly with peak CRP and additive change, but not with baseline CRP although the effect was of similar magnitude. There was no independent association of wound length or ethnic group with the exception of a low baseline CRP in the small group of subjects from the ’other’ ethnic group. There were no significant associations of social class with any of the three measures of CRP, nor of being on anti-hypertensive medication.

**Table 3:** Multivariate analysis of the predictors of baseline, peak CRP and additive change in CRP following surgery

	**Baseline CRP (proportional difference)**	**Peak CRP (proportional difference)**	**Difference in peak CRP peak and baseline(proportional difference)**
Baseline CRP (per 10 fold increase)		1.77 (1.25-2.52) P = 0.002	0.85 (0.58-1.26)
Waist:hip ratio (per 10% increase)	1.43 (1.02- 2.01) p = 0.04	1.46 (1.14-1,88) P = 0.002	1.41 (1.06- 1.59) P = 0.02
Age (per decade)	1.20 (0.81-1.56)	1.27 (1.00-1.62) p = 0.05	1.20 (0.92-1.57)
Duration of surgery (per minute)	1.05 (0.99-1.10) p = 0.06	1.05 (1.02-1.09) P = 0.007	1.06 (1.02-1.11) p = 0.004
Wound length (per cm)	1.17 (0.83-1.64)	0.94 (0.73-1.21)	1.07 (0.81-1.41)
Ethnic Group: n, %			
White Caucasian	1.30 (0.77-2.20)	1.15 (0.78-1.69)	1.11 (0.71-1.71)
Afro Carib	1.52 (0.81-2.87)	0.88 (0.55-1.41)	0.99 (0.59-1.68)
Asian	1.91 (0.78-4.68)	1.04 (0.53-2.03)	1.26 (0.60-2.67)
Other (reference)	0.27 p = 0.04	0.96	0.72
Smoking status (%)			
Current (reference)	0.97	0.96	0.96
Ex	1.17(0.47-1.38)	1.00 (0.75-1.33)	0.96 (0.70-1.33)
Never	0.89(0.62-1.26)	1.04 (0.81-1.35)	1.08 (0.81-1.45)
Exercise			
<2 x /week (reference) 2 + x/week	0.80(0.47-1.38)	1.27 (0.86-1.89)	1.39 (0.89-2.17)
Occupation			
Manual	0.96 (0.58-1.56)	0.90 (0.62-1.29)	0.84(0.56-1.26)
Non-manual	0.83 (0.54-1.26)	1.39 (1.02-1.89)	1.29(0.91-1.82)
Retired (reference)	1.27	0.81	0.93
Anti-hypertensive medication	1.12 (0.59-2.12)	1.34 (0.83-2.19)	1.47(0.91-2.40)

All predictors are mutually adjusted. The analysis was by ANOVA.

When waist:hip ratio and waist circumference were both put into the full multivariate model, waist:hip ratio retained its statistical significance and the magnitude of the effect was little diminished- 1.40(1.00-1.96) p = 0.05, whereas the effect of waist circumference was abolished- 1.07 (0.81-1.43) NS.

**Figure 2:**
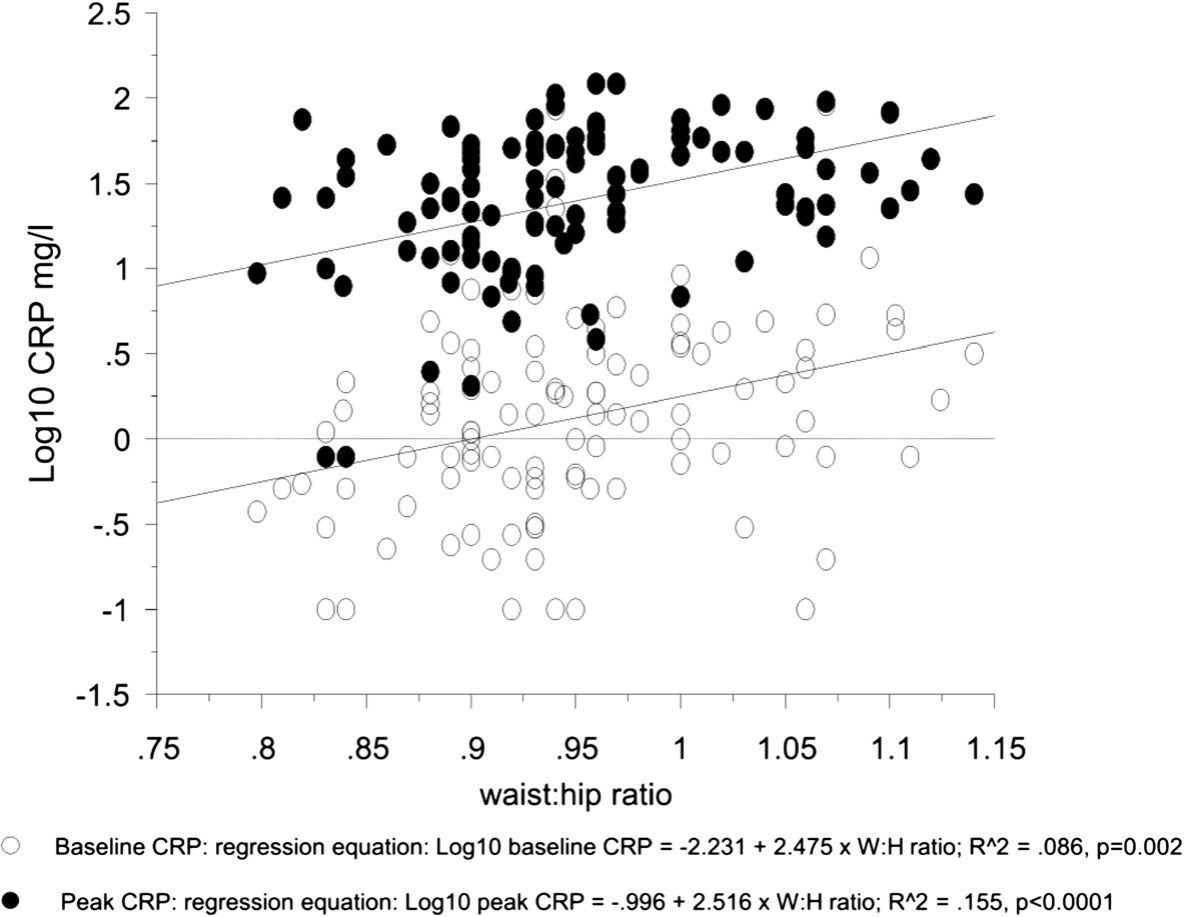
The regression lines for the association of waist:hip ratio with baseline and peak CRP.

Table 4 shows the effect sizes of age, baseline CRP, waist:hip ratio, duration of surgery and wound length mutually adjusted in a model restricted to the independent predictors with the exception of wound length which was included because of it’s importance as a potential confounder. As all these variables are continuous the model was run using multiple regression. Duration of surgery and wound length were not included in the model of baseline CRP. The magnitude of the effect of waist:hip on baseline, adjusted peak and difference in CRP is little changed by adjustment for the additional factors and also striking is the similarity of the magnitude of the effects on all three measures of CRP. This is illustrated in Figure 2 where it is demonstrated that the scatter plots for waist:hip ratio versus CRP at baseline and peak have parallel regression lines and that scatter of points seen at baseline is reduced as the dominant influence of surgery on levels manifests.

**Table 4:** Multiple regression model of the key explanatory variables mutually adjusted

	**Baseline CRP (proportional difference)**	**Peak CRP (proportional difference)**	**Difference in peak CRP peak and baseline(proportional difference)**
Baseline CRP (per 10 fold increase)		1.72 (1.23-2.40) p = 0,002	0.86 (0.59-1.25)
Waist:hip ratio(per 10% increase)	1.46 (1.01-2.12) p = 0.04	1.53 (1.17-2.01) p = 0.002)	1.47 (1.08-1.98) p = 0.01
Age (per decade)	1.28 (1.05-1.56) p = 0.01	1.15 (1.00-1.33) p = 0.05	1.13 (0.96-1.32) p = 0.1
Duration of surgery (per minute)	1.05 (1.01-1.10) p = 0.03	1.05 (1.01-1.08) p = 0.007	1.06 (1.02-1.10) p = 0.005
Wound length (per cm)	1.10 (0.79-1.53)	0.99 (0.78-1.25)	1.08 (0.83-1.41)

Table 5 shows the relationship of waist;hip ratio to baseline characteristics. The only important relationships were with age and manual social class as would be expected. There was no association with duration of surgery, but there was an association with wound length as might be expected.

**Table 5:** Relation of waist:hip ratio to baseline risk factors. Statistical testing for categorical variables was by ANOVA and linear regression for continuous variables

	**Waist:hip ratio proportional change**
Age (per decade)	1.49 (1.17- 1.90) p = 0.0013
Duration of surgery(per minute)	1.02 (0.97- 1.09)
Wound length (per cm)	1.47 (1.14- 1.88) p = 0.003
Ethnic Group: n, %	
White Caucasian	0.81 (0.42- 1.58)
Afro Carib	0.44 (0.18- 1.07)
Asian	1.52 (0.47- 4.86)
Other (reference)	1.85
Smoking status (%)	
Current (reference)	0.79
Ex	1.34 (0.78-2.27)
Never	0.94 (0.59-1.50)
Exercise	
2 + ×/week v < 2× /week	0.85 (0.39- 1.84)
Occupation	
Manual (reference)	1.66 (NS)
Non-manual	0.82 (0.49-1.40)
Retired	0.73 ( 0.40-1.33)

## Discussion

This is the first study to examine the effect of abdominal adiposity and other environmental risk factors on the inflammatory response to injury as indicated by serum CRP. We primarily defined CRP response to surgery as the logarithm of postoperative peak CRP adjusted for preoperative CRP level. We also used a secondary definition of the logarithm of the additive change in CRP pre and post surgery. These two measures gave similar results. CRP response was higher with higher waist:hip ratio, increasing age and longer duration of surgery. The relation to waist:hip ratio was continuous with no apparent threshold effect.

Although some of the established risk factors for atherosclerosis and other chronic diseases such as cigarette smoking, ethnic group and social class exhibited associations with baseline CRP (for exercise there was a trend in the expected direction) in univariate analysis, there were no important effects on response after adjustment for other factors. The study was not designed to explore these effects and may have been too small to detect them or alternatively they may not have been present. The reduction in the magnitude of the effects supports the latter.

This is a relatively small study, but the magnitude of the effects we observed for waist:hip ratio leave little doubt as to the fact that they are true findings. We chose hernia repair as it is easily standardised, easy to grade the insult, and is associated with a demonstrable inflammatory response. It is unlikely that the exaggerated response in subjects with a high waist:hip ratio and increased age were due to differences in surgical technique, as the association was independent of duration of surgery or wound length even although the latter was associated with waist:hip ratio. We cannot entirely discount the possibility that the increased response with waist:hip ratio was due to increased damage to subcutaneous adipose tissue due to a greater depth of incision. However if this were the case, it would be expected that wound length would be independently associated with inflammatory response and that it would confound the association with waist:hip ratio which it did not. It seems unlikely that wound depth which we did not measure would vary inversely with wound length. Importantly, there was no association of the duration of surgery with waist:hip ratio. Furthermore, the association of waist:hip ratios with CRP response was continuous throughout the range of ages and waist:hip ratios with no evidence of a threshold for the effect. Likewise it seems unlikely that differences in CRP response could be explained by post-operative infection being more common as waist:hip ratio rises. We did not find any instances of post-operative infection and if lung infection caused by atelectasis related to the anaesthetic were responsible, a higher post-operative response in smokers would have been expected. It seems most likely therefore that waist:hip ratio is associated with an increased CRP responsiveness to injury through mechanisms independent of the degree of tissue damage or procedure related infection.

The C-reactive protein response to inguinal hernia repair has been described on numerous occasions, but with the goal of comparing operative technique rather than studying host characteristics which determine the inflammatory response [14]. Future studies in this area should take account of age and waist:hip ratio. They should also take account of the length of incision and more importantly the duration of surgery. One other study has explored the relationship of obesity to the post operative inflammatory response following laparoscopic cholycystectomy and like the current study found it to be exagerated with impaired anti-inflammatory cytokine production. This study however was small with only 34 subjects, only compared obese and normal BMI subjects and did not study other host characteristics which could influence the inflammatory response [13].

We chose peak CRP adjusted for baseline CRP as the measure of response. Similar findings were obtained when the additive change in CRP was used. However the better model fit was with the former. Whilst baseline CRP was strongly associated with peak CRP, there was no association with the additive change, suggesting that regression to the mean is not an explanation for association of waist:hip ratio with the latter. For logistic and resource reasons we did not measure CRP beyond 48 hrs and it is possible that we would have obtained some higher peak values had we done so. However, values beyond 48 hrs would have been more likely to be influenced by factors other than the initial surgical insult which it was our goal to measure the effect of.

The measure of adiposity most strongly related to CRP response was waist:hip ratio, which is the best measure of visceral adipose tissue, followed by waist circumference with only a weak association with BMI. Had we measured visceral adipose tissue more directly using CT scanning, it is likely that we would have found an even stronger association. The stronger association of waist:hip ratio is in line with other studies assessing the association of adiposity with circulating inflammatory markers and the metabolic syndrome [6,8]. Studies of the association of adiposity with disease end points however suggest that BMI may be the better marker [2], but that waist:hip ratio has an additional effect independent of BMI. BMI is the more imperfect measure of abdominal adiposity but possibly a better measure of total adiposity. It is also easier to measure than waist:hip ratio in large field studies.

It can be speculated that the finding that waist:hip ratio and age influence baseline and post-injury CRP levels with a very similar size of effect supports the contention that baseline CRP is a measure of inflammatory responsiveness to casual stimuli and that obesity and higher age modulate the generic excitability of the inflammatory system leading to both higher baseline CRP and higher CRP response to surgery through similar mechanisms. The mechanism for the association of baseline CRP and waist:hip ratio to cardiovascular disease and other disease outcomes could be through this increase in inflammatory system excitability leading to exaggerated local inflammatory responses to environmental exposures, specifically hypertension at the blood vessel wall in the case of atherosclerosis, and various organ specific locations in cancer.

There are other possible explanations for the association of basal CRP with risk of atherosclerosis rather than as an indicator of immune responsiveness. The elevated CRP could be the result of inflammation in response to sub-clinical disease. This however is unlikely, as the relationship of baseline CRP with many adverse outcomes does not diminish with time from when it was measured [1]. Another interpretation is that the environmental factors which cause disease through other mechanisms also activate inflammation. We cannot discount this possibility. Finally it is possible that the inflammatory responses of which CRP is a marker induce changes in conventional risk factors such as lipids, insulin and blood sugar which are related to insulin resistance as part of the metabolic syndrome and that it is these which result in the adverse consequences. Only one other study has addressed this issue, the Rancho Bernado Study [15]. In this study, adjustment for CRP attenuated the effect of the metabolic syndrome by 35%, whereas adjustment of the effect of CRP for the metabolic syndrome did not reduce the size of effect. This suggests that the association between features of the metabolic syndrome and atherosclerosis may at least to an important degree be explained by its association with underlying inflammatory mechanisms.

As well as the relevance of our findings to the study of the inflammatory response to surgery, atherosclerosis, cancer and other chronic diseases, these findings would explain the rather puzzling observation that obesity, increased waist:hip ratio and CRP are related to risk of death from external causes and the higher rates of morbidity in the obese from trauma, surgery and acute pancreatitis [16,17]. Furthermore it would explain the higher rates of osteoarthritis in non weight bearing joints in obese subjects [18]. Exaggerated inflammatory responses could be damaging through a number of mechanisms and could delay healing and resolution of inflammation.

## Conclusions and future work

When comparing the invasiveness of surgical methods using CRP levels, waist:hip ratio and age need to be taken into consideration. Higher CRP levels could be due to higher waist:hip ratios and age rather than the more invasive procedure. The duration of surgery also needs to be considered as an important factor.

It can also be speculated that WHR may modulate the generic excitability of the inflammatory system leading to higher baseline and higher CRP responses to injury through similar but as yet to be determined mechanisms. This could increase the risk of atherosclerosis and other chronic diseases in response to environmental insults.

Larger studies are called for to assess other possible determinants of inflammatory responses to injury such as blood lipid levels, as well as the effect of drugs which reduce risk of death from chronic disease such as statins and aspirin. These drugs could possibly reduce the risk associated with surgery. The effect of obesity and other factors on the response of other cytokines to standardised injury is also warranted. We propose unilateral hernia repair as a useful model for studying these effects.

This work was supported by the Croydon University Health R&D Fund and the St George’s Special Trustees.

## Competing interests

The authors declare that they have no competing interests.

## Authors’ contributions

SI helped design and acquired the data, DG performed laboratory analyses and critically revised the paper, BU performed all the surgery and critically reviewed the manuscript, DK made a substantial contribution to analysis and interpretation of the data and critically revised the manuscript, MM conceived of and designed the study and played an important role in data analysis and writing of the manuscript. All authors read and approved the final manuscript.
